# Total mercury exposure in early pregnancy has no adverse association with scholastic ability of the offspring particularly if the mother eats fish

**DOI:** 10.1016/j.envint.2018.03.024

**Published:** 2018-07

**Authors:** Joseph Hibbeln, Steven Gregory, Yasmin Iles-Caven, Caroline M. Taylor, Alan Emond, Jean Golding

**Affiliations:** a Section on Nutritional Neurosciences, Laboratory of Membrane Biophysics & Biochemistry, Nat. Institutes on Alcohol Abuse & Alcoholism, NIH, 31 Center Drive 1B/58, Bethesda, MD 20892, USA; b Centre for Child and Adolescent Health, Bristol Medical School (Public Health Sciences) University of Bristol, Oakfield House, Oakfield Grove, Bristol BS8 2BN, UK

**Keywords:** ALSPAC, Avon Longitudinal Study of Parents & Children, CDC, Centers for Disease Control & Prevention, LOD, Level of detection, IQR, Interquartile range, NARA, Neale Analysis of Reading Ability, TOWRE, Test of Word Reading Ability, WISC, Wechsler Intelligence Scale for Children, WORD, Wechsler Objective Reading Dimension, WPPSI, Wechsler Preschool and Primary Scale of Intelligence, ALSPAC, Prenatal mercury, Prenatal fish consumption, Reading, Mathematics, Science

## Abstract

There is a public perception that relatively low doses of mercury found in seafood are harmful to the fetal brain but little consistent evidence to support this. In earlier publications we have shown no adverse associations between maternal total blood mercury levels and child behaviour, early development or cognitive function as measured by IQ. However, for IQ the lack of adverse association was conditional upon the mother being a fish eater.

In this paper we analyse further data from the Avon Longitudinal Study of Parents and Children (ALSPAC), this time examining whether prenatal exposure to total mercury is associated with the child's scholastic abilities in reading, spelling, phoneme awareness, mathematics and science; the number of participants with prenatal mercury and relevant test results varied from 1500 to 2200. Multiple regression was used to assess relationships between prenatal total blood mercury concentrations and 16 different test results, after taking account of a variety of potential confounders; in parallel, logistic regression was used to determine associations with the risk of the child being in the lowest 15% of each score. Analyses were repeated stratifying for fish consumption and sex of the child.

There was no evidence of harm associated with the level of total mercury, provided the mother ate fish during pregnancy. This was particularly true for tests of mathematics and science. We conclude that women should be confident that eating fish in pregnancy is beneficial for their unborn child.

## Highlights

• High levels of maternal blood Hg may result in neurological defects in the child.
• We examined lower prenatal Hg levels and offspring educational.
• No deleterious effects were found for reading or spelling results.
• Math/scientific skills were unaffected by maternal Hg level provided she ate fish.

## 1 Introduction

Very high doses of total mercury in pregnancy have a harmful effect on the development of offspring, with increased risks of cerebral palsy and cognitive impairment (Snyder, 1971). Such deleterious exposures have been found, for example, when spillages of pollutants into water have undergone bioaccumulation by fish and shellfish, with subsequent consumption by the population (such as in Minamata in Japan), or when grain treated with mercury was unintentionally put into the human food chain (such as happened in Iraq) (Amin-Zaki et al., 1976). Such adverse events have understandably raised concerns about all levels of exposure to mercury during pregnancy.

There have been several studies measuring maternal prenatal exposure to low levels of mercury and subsequent child development, with results that are sometimes reported erroneously as causing problems to the offspring (e.g. Myers et al., 2015). Such studies necessitate collecting data during pregnancy and then following the offspring into childhood (and beyond). Using a large population cohort in the UK, we have found that early child development and IQ measured at age 8 years were not affected by the mother's total mercury level if she ate fish (Golding et al., 2016a, Golding et al., 2016b, Golding et al., 2017).

The most important outcome of mercury exposure from the point of view of the future economic success of a country lies in the ability of its workforce, particularly regarding their literacy and numeracy. However, to our knowledge, only two studies appear to have considered these outcomes: the Seychelles Child Development Study (Davidson et al., 2010, Davidson et al., 2011) and a study in New Zealand (Gearhart et al., 1995).

The New Zealand study is little known; details of the methodology used are scanty, and it is not clear that all the publications arising from it have been peer-reviewed. The study started in the North Island of New Zealand with a birth survey of 10,970 pregnancies. A small group of women with high hair mercury levels who were relatively high fish consumers (>3 times per week) were identified and each was matched with three controls of differing mercury levels and fish consumption. The offspring of 57 matched sets were followed up at 6 years of age and given a battery of 26 tests which included reading and mathematics. Although the authors did not give the results for each test, they did claim that none showed an association between maternal mercury level and outcome. However, if an outlier of over 50 ppm higher than the rest of the cohort was removed, there were six of the outcomes that were associated at the 10% level, but it is unclear which test results were implicated (Crump et al., 1998).

In comparison, the longitudinal study undertaken in the Seychelles is very well documented and well designed. A study of 643 unselected children in this cohort had results from their national standardised examinations at ages 9 and 17 years linked to their mother's prenatal mercury level (as estimated from maternal hair). The subjects of the examinations undertaken at 9 years of age comprised English, French, Creole, mathematics, science and social studies; at 17 years geography and history were added but Creole and social studies were excluded. None of these outcomes showed any adverse relationship with prenatal mercury exposure, and there was a beneficial association with one of the math tests at 17 years (Davidson et al., 2010, Davidson et al., 2011).

The study in the Seychelles was undertaken because the population of the archipelago consumed a large quantity of fish on average and therefore, if there were adverse effects of such a diet, particularly regarding the consequent increased levels of mercury, this study should have sufficient statistical power to reveal it. However, despite a large variety of measures over the years, there have been no significant adverse outcomes attributable to mercury. Indeed comparison of the scholastic abilities of the Seychelles children with other countries in Africa and the Indian Ocean shows them to be among the most advanced in ability (Leste and Davidson, 2004).

Nevertheless, it is important that similar studies be undertaken in areas where less fish is consumed, and where any adverse effects of prenatal mercury exposure may be masked by beneficial effects of fish but revealed in the offspring of women who have not eaten fish. We therefore used the comprehensive data collected on a population of pregnant women in the UK in 1991–2, whose total blood mercury level is available for the first half of pregnancy and whose offspring have been followed throughout childhood and adolescence.

## 2 Material and methods

### 2.1 The study design

The Avon Longitudinal Study of Parents and Children (ALSPAC) aimed to study all births to women resident in a geographic area (Avon) in the UK, whose expected date of delivery lay between the 1st April 1991 and 31st December 1992. It recruited 14,541 women who completed at least one questionnaire. Of these initial pregnancies, there was a total of 14,676 fetuses, resulting in 14,062 live births and 13,988 children who were alive at one year of age. The study's stated aims were to determine ways in which the individual's genotype combines with environmental pressures to influence health and development. It recognised the need to identify environmental factors prospectively during pregnancy. The advantage of an area-based study concerned the relative ease of contacting the pregnant women, collecting biological samples and providing facilities for hands-on examination of the study children under controlled circumstances. (Boyd et al., 2013; Golding et al., 2001). For full details of all the data collected see the study website: www.bristol.ac.uk/alspac/researchers/data-access/data-dictionary/. Ethical approval for the study was obtained from the ALSPAC Ethics and Law Committee and the Local Research Ethics Committees.

### 2.2 The exposures

#### 2.2.1 Measurement of total blood mercury

This has been described in detail elsewhere (Golding et al., 2013). In brief, blood samples were collected in acid-washed vacutainers provided by ALSPAC to midwives who were collecting blood for clinical purposes on the first occasion on which they saw the pregnant women. Samples were kept as whole blood in the original tubes for long-term storage at 4 °C. After approximately 19 years, the samples were sent to the Centers for Disease Control and Prevention (CDC) for analysis of whole blood mercury, lead, selenium, and cadmium (CDC method 3009.1). Of the 4484 samples collected, 4131 had valid results for total mercury. Only three samples had total mercury values below the assay limit of detection (LOD) (0.24 μg/L); they were assigned the LOD value divided by the square root of 2.

Gestational age at sample collection ranged from 1 to 42 weeks, with a median value of 11 weeks and mode of 10 weeks. The IQR (inter-quartile range) was 9 to 13 weeks, and 93% of the samples were collected at <18 weeks of gestation.

#### 2.2.2 Maternal consumption of fish

The pregnant woman was sent a questionnaire at 32 weeks gestation, which included a food frequency questionnaire. This enquired about the frequency with which she ate white fish and oily fish. We used these two questions to identify women who ate no fish, as previously described (Golding et al., 2016a, Golding et al., 2016b, Golding et al., 2017).

### 2.3 The outcome measures

A total of 15 different scholastic tests have been used in this paper, covering spelling, reading, phoneme understanding, mathematics and science; 11 of these tests were administered in the ALSPAC clinics in a one-to-one situation, and the three mathematics reasoning and the one scientific reasoning tests were administered in a school setting. Details of the tests used are described in the Supplementary Information.

### 2.4 The analyses

Ways in which the total blood mercury varies with demographic and lifestyle factors is shown in Appendix Table 1. Because of these and other associations we therefore took account of maternal age at the child's birth, parity of the mother at the birth (no. previous births: 0 v 1+); maternal education level (in five levels of achievement); housing tenure (owner occupied; public housing; other rented); level of household crowding (no. persons in the household divided by the number of living rooms and bedrooms); no. of stressful life events during the first half of pregnancy; whether the mother smoked at mid-pregnancy (yes; no); whether any alcohol was drunk mid-pregnancy (yes; no); whether the infant was breast fed (yes; no); and the family adversity index (a score comprised of a number of adverse features present during pregnancy including presence of maternal depression and anxiety). Because the educational ability of the child depends on the length of time he/she has attended school, we took account of that rather than age when tested.

We mainly employed regression analyses, treating both mercury and the scholastic measures as continuous variables; results for the adjusted and unadjusted outcomes are presented as β coefficients (i.e. the change in value per SD of total mercury). The analyses were repeated according to whether the mother had eaten fish or not prenatally, and whether the child was a boy or girl.

To determine whether the children who had low results on the scholastic tests were at especial risk, we used logistic regression to assess the variation of total mercury levels with the binary outcome concerning whether the child was in the lowest 15% of the relevant distribution. Both sets of analyses took account of the 11 possible confounders listed above. The analyses were carried out using STATA version 14 (StataCorp LLC). Since we do not consider that the data are likely to be missing at random, we have not included analyses concerning missingness.

The total numbers of children for whom there were both maternal prenatal total mercury levels and an academic outcome vary from 1569 to 2224. Subdividing by whether the mother ate fish, and by the sex of the offspring resulted in minimum numbers of 120–121 for the non-fish consuming population. Consequently, for the regression analyses the statistical power for these subdivisions ranges from being 80% sure of a statistically significant result from approximately 0.18 SDs for the fish eaters, and 0.27 SDs for the non-fish eaters.

## 3 Results

The basic statistics for each of the outcome measures are given in Table 1. This table also shows the available data for children whose mothers ate fish in pregnancy and those that did not. These unadjusted data indicate that, except for arithmetic, each test score is higher for children whose mothers ate fish compared with the results for the children whose mothers ate no fish.

**Table 1:** The basic data for the spelling, reading and phoneme tests for (i) all children; (ii) children whose mothers at fish in pregnancy, and (iii) children whose mothers ate no fish in pregnancy.

Outcome	All children		Mother ate fish		Mother ate no fish	
Measure	N	Mean (95% CI)	N	Mean (95% CI)	N	Mean (95% CI)
Spelling						
7 year	2192	7.7 (7.5, 7.9)	1815	7.9 (7.7, 8.1)	268	7.0 (6.5, 7.6)
9 year	2117	10.3 (10.1, 10.4)	1748	10.4 (10.2, 10.5)	253	9.9 (9.4, 10.3)

Word reading test						
7 year	2224	28.2 (27.8, 28.6)	1842	28.5 (28.1, 28.9)	271	27.0 (25.8, 28.2)
9 year	2121	7.6 (7.5, 7.7)	1752	7.7 (7.5, 7.8)	253	7.2 (6.9, 7.6)

Reading comprehension						
9 Year	2079	100.8 (100.3, 101.3)	1724	101.3 (100.8, 101.9)	242	99.1 (97.5, 100.7)

Reading speed						
9 Year	2073	105.8 (105.3, 106.3)	1721	106.3 (105.7, 106.8)	240	104.8 (103.2, 106.4)

Reading accuracy						
9 year	2079	104.7 (104.1, 105.3)	1724	105.2 (104.6, 105.9)	242	102.8 (100.9, 104.7)

Reading fluency						
13 year	1574	82.7 (82.2, 83.2)	1313	83.0 (82.5, 83.6)	182	81.4 (79.8, 83.1)

Phoneme tests						
7 year	2223	19.9 (19.5, 20.3)	1842	20.2 (19.8, 20.6)	271	18.6 (17.4, 19.8)
9 year	2109	5.3 (5.2, 5.4)	1743	5.4 (5.3, 5.5)	251	5.0 (4.7, 5.3)

Arithmetic						
Age 8	2067	10.6 (10.4, 10.8)	1732	10.6 (10.4, 10.8)	234	10.7 (10.2, 11.2)

Mathematics reasoning						
SY4	1212	11.0 (10.9, 11.2)	919	11.2 (11.0, 11.4)	167	10.6 (10.1, 11.2)
SY6	2041	19.4 (19.0, 19.7)	1567	19.9 (19.6, 20.3)	275	18.0 (17.2, 18.8)
SY8	896	23.3 (22.9, 23.8)	778	23.9 (23.4, 24.4)	118	21.5 (20.0, 23.0)

Scientific reasoning						
SY6	2016	6.0 (5.9, 6.1)	1537	6.3 (6.1, 6.4)	281	5.4 (5.1, 5.7)

### 3.1 Overall association of prenatal total mercury levels with academic outcomes in the offspring

The unadjusted data of Table 2A provide the regression coefficients of each test per standard deviation (SD) of prenatal mercury. There is a significant positive association for each of the 15 unadjusted educational outcomes with the maternal total mercury level. After adjustment, however, there were no significant associations, although most of the regression coefficients remained positive, and the relationship with scientific understanding was weakly significant (*P* = 0.054). When the children with the poorer 15% of the test results were examined using logistic regression, there were similar findings – there were apparently protective effects with increasing total mercury levels which became non-significant on adjustment (Table 2B).

**Table 2A:** Unadjusted and adjusted^a^ associations of prenatal total blood mercury with measures of scholastic ability using multiple regression.

Outcome	Unadjusted			Adjusted		
Measure	N	β(95% CI)	P	N	β(95% CI)	P
Spelling						
7 years	2192	+0.31 (+0.14,+0.47)	<0.001	1863	+0.02 (−0.16,+0.20)	0.833
9 years	2117	+0.21 (+0.08, +0.35)	0.002	1794	−0.05 (−0.20,0.10)	0.494

Word reading						
7 years	2224	+0.81 (+0.46,+1.15)	<0.001	1888	+0.09 (−0.30,+48)	0.646
9 years	2121	+0.19 (+0.09,+0.29)	<0.001	1797	−0.01 (−0.11,+0.10)	0.895

Reading comprehension						
9 years	2079	+0.71 (+0.41,+1.01)	<0.001	1765	−0.16 (−1.48,+1.15)	0.307

Reading speed						
9 years	2073	+2.65 (+1.62,+3.68)	<0.0001	1761	+0.31 (−0.80,+1.42)	0.588

Reading accuracy						
9 years	2079	+1.54 (+0.75,+2.33)	<0.0001	1765	−0.28 (−1.13,+0.57)	0.518

Reading fluency						
13 years	1574	+0.85 (+0.40,+1.29)	<0.001	1363	+0.22 (−0.26,+0.71)	0.362

Phoneme tests						
7 years	2223	+0.62 (+0.26,+0.97)	0.001	1888	+0.10 (−0.29,+0.50)	0.606
9 years	2109	+0.14 (+0.05,+0.24)	0.004	1788	−0.00 (−0.11,+0.10)	0.937

Arithmetic						
8 years	2067	+0.28 (+0.12,+0.44)	0.001	1762	+0.03 (−0.15,+0.21)	0.716

Mathematics reasoning						
SY4	1213	+0.41 (+0.24,+0.58)	<0.001	943	+0.09 (−0.11,+0.29)	0.392
SY6	2041	+0.86 (+0.58,+1.13)	<0.001	1578	+0.06 (−0.26,+0.38)	0.721
SY8	986	+1.14 (+0.70,+1.57)	<0.001	779	+0.29 (−0.17,+0.75)	0.223

Scientific reasoning						
SY6	2016	+0.36 (+0.26,+0.47)	<0.0001	1585	+0.12 (−0.00,+0.27)	0.054

SY = School year.

Regression coefficients of each test represent change in score per standard deviation (SD) of prenatal mercury.

a Adjusted for month of birth relative to school year, sex of child, maternal age, parity, maternal education, family adversity index, housing tenure, household crowding, prenatal life events, prenatal smoking, prenatal alcohol.

**Table 2B:** Unadjusted and adjusted^a^ associations of prenatal total blood mercury with being in the lowest 15% of measures of scholastic ability, using logistic regression.

Outcome	Unadjusted			Adjusted		
Measure	N	OR (95% CI)	P	N	OR (95% CI)	P
Spelling						
7 years	2192	0.78 (0.69, 0.88)	0.0001	1894	0.94 (0.83, 1.08)	0.408
9 years	2117	0.89 (0.79, 0.99)	0.041	1829	1.02 (0.90,1.16)	0.734

Word reading						
7 years	2224	0.82 (0.73, 0.92)	0.001	1920	0.98 (0.85, 1.12)	0.734
9 years	2121	0.81 (0.71, 0.92)	0.002	1832	1.02 (0.88, 1.17)	0.838

Reading comprehension						
9 years	2079	0.82 (0.72, 0.93)	0.003	1799	1.03 (0.90, 1.19)	0.648

Reading speed						
9 years	2073	0.81 (0.72, 0.92)	0.001	1795	0.98 (0.85, 1.13)	0.808

Reading accuracy						
9 years	2079	0.81 (0.71, 0.92)	0.001	1799	0.99 (0.86, 1.14)	0.900

Reading fluency						
13 years	1574	0.87 (0.77, 0.99)	0.047	1384	0.99 (0.86, 1.14)	0.880

Phoneme tests						
7 years	2223	0.86 (0.76, 0.96)	0.009	1920	0.97 (0.85, 1.10)	0.630
9 years	2109	0.84 (0.74, 0.95)	0.004	1827	0.95 (0.83, 1.08)	0.416

Arithmetic						
8 years	2067	0.87 (0.77, 0.98)	0.025	1791	1.03 (0.90, 1.18)	0.686

Mathematics reasoning						
SY4	1212	0.77 (0.64, 0.93)	0.006	947	0.88 (0.69, 1.11)	0.277
SY6	2041	0.78 (0.69, 0.89)	<0.0001	1609	1.04 (0.89, 1.20)	0.658
SY8	986	0.64 (0.51,0.80)	<0.0001	791	0.87 (0.69,1.11)	0.267

Scientific reasoning						
SY6	2016	0.79 (0.68, 0.92)	0.003	1585	0.97 (0.81, 1.17)	0.747

SY = School year.

Odds ratios for each test represent odds of being in the lowest 15% of the test score.

a Adjusted for month of birth relative to school year, sex of child, maternal age, parity, maternal education, family adversity index, housing tenure, household crowding, prenatal life events, prenatal smoking, prenatal alcohol.

### 3.2 Comparison of fish and non-fish eaters

Regressing each of the academic outcomes on maternal total mercury levels after adjustment (Table 3A) demonstrated no significant associations for either the children of the fish eaters or of the non-fish eaters. However, there was evidence of a significant difference between the regression coefficients for the science reasoning in school year 6 and the mathematics comprehension tests in school years 6 and 8 (as illustrated by comparison of the confidence intervals between the associations between mercury and outcome among the fish and non-fish eaters). These showed evidence that the maternal mercury level was associated negatively with the results on these tests if the mother had not eaten fish in pregnancy - in contrast to the children whose mothers had eaten fish for whom there were positive associations with total mercury.

**Table 3A:** Adjusted^a^ associations (β(95%CI)) of prenatal total blood mercury with measures of scholastic ability, analysed using multiple regression, stratified according to whether the mother had eaten fish during pregnancy.

Outcome	Mother ate fish			Mother ate no fish		
Measure	N	β (95% CI)	P	N	β (95% CI)	P
Spelling						
7 Year	1628	+0.03 (−0.17,+0.22)	0.790	231	−0.39 (−1.16,+0.38)	0.317
9 Year	1578	−0.05 (−0.20,+0.11)	0.553	210	−0.02 (−0.65,+0.60)	0.940

Word reading test						
7 Year	1651	+0.09 (−0.30,+0.49)	0.639	233	−0.56 (−2.18,+1.07)	0.501
9 Year	1581	−0.00 (−0.11,+0.11)	0.996	210	+0.09 (−0.36,+0.55)	0.579

Reading comprehension						
9 Year	1558	−0.16 (−0.49,+0.17)	0.336	201	−0.23(−1.68,+1.23)	0.757

Reading speed						
9 Year	1555	+0.54 (−0.63,+1.72)	0.364	200	−0.77 (−5.58,+4.04)	0.752

Reading accuracy						
9 Year	1558	−0.37 (−1.26,+0.52)	0.412	201	+1.35 (−2.57,+5.27)	0.497

Reading fluency						
13 Year	1202	+0.16 (−0.35,+0.66)	0.547	156	+0.87 (−1.22,+2.97)	0.412

Phoneme tests						
7 Year	1651	+0.14 (−0.28,+0.56)	0.527	233	−0.46 (−2.16,+1.23)	0.590
9 Year	1573	+0.00 (−0.11,+0.12)	0.944	209	−0.03 (−0.47,+0.41)	0.883

Arithmetic						
8 Years	1557	+0.08 (−0.11,+0.27)	0.414	200	−0.07 (−0.80,+0.68)	0.860

Mathematics reasoning						
SY4	802	+0.16 (−0.05,+0.38)	0.141	136	−0.48 (−1.27,+0.30)	0.224
SY6	1357	+0.13 (−0.21,+0.48)	0.450	212	−0.96 (−2.05,+0.13)	0.083^b^
SY8	676	+0.21 (−0.29,+0.71)	0.415	100	−0.58 (−0.96,+0.21)	0.457^b^

Scientific reasoning						
SY6	1348	+0.14 (+0.01,+1.12)	0.042	229	−0.29 (−0.68, +0.10)	0.149^b^

SY = School year.

Regression coefficients of each test represent change in score per standard deviation (SD) of prenatal.

a Adjusted for month of birth relative to school year, sex of child, maternal age, parity, maternal education, family adversity index, housing tenure, household crowding, prenatal life events, prenatal smoking, prenatal alcohol.

b Significant difference between children of mothers who ate fish and those whose mothers did not.

Similarly, when the lowest 15% of each score were considered, after adjustment (Table 3B) there were no significant associations, but there was, again, evidence of an interaction between results contingent upon the fish-eating of the mother prenatally. For example, the child of the fish eater was less likely to be in the lowest 15% of mathematics in year 6, and of science reasoning in year 6, whereas the child of the non-fish eater was more at risk of such outcomes.

**Table 3B:** Adjusted^b^ associations (β(95%CI)) of prenatal total blood mercury with measures of scholastic ability according to whether the mother had eaten fish during pregnancy; using logistic regression analyses.

Outcome	Mother ate fish			Mother ate no fish		
Measure	N	OR (95% CI)	P	N	OR (95% CI)	P
Spelling						
7 year	1651	0.96 (0.83, 1.11)	0.569	239	0.89 (0.52, 1.50)	0.652
9 year	1603	1.03 (0.90, 1.18)	0.688	220	1.07 (0.65, 1.76)	0.790

Word reading test						
7 year	1675	0.98 (0.85, 1.14)	0.819	241	1.12 (0.68, 1.83)	0.654
9 year	1606	1.01 (0.87, 1.17)	0.914	220	1.07 (0.58, 1.98)	0.835

Reading comprehension						
9 year	1583	1.05 (0.90, 1.22)	0.550	210	1.06(0.61, 1.85)	0.838

Reading speed						
9 year	1580	0.98 (0.84, 1.13)	0.746	209	1.17 (0.69, 2.01)	0.559

Reading accuracy						
9 year	1583	1.02 (0.88, 1.17)	0.834	210	0.81 (0.46, 1.43)	0.471

Reading fluency						
13 year	1218	1.04 (0.90, 1.21)	0.574	161	0.81 (0.47, 1.41)	0.459

Phoneme tests						
7 year	1675	0.98 (0.86, 1.13)	0.790	241	1.18 (0.76, 1.85)	0.461
9 year	1603	0.94 (0.82, 1.09)	0.406	218	0.94 (0.53, 1.64)	0.821

Arithmetic						
8 years	1578	1.01 (0.87, 1.17)	0.877	208	0.81 (0.40, 1.65)	0.558

Mathematics reasoning						
SY4	804	0.81 (0.62, 1.07)	0.139	136	1.38 (0.60, 3.13)	0.447
SY6	1377	0.97 (0.82, 1.15)	0.720	223	1.59 (0.96, 2.62)	0.071^a^
SY8	686	0.87 (0.66, 1.15)	0.328	102	0.79 (0.44, 1.44)	0.450

Scientific reasoning						
SY6	1348	0.91 (0.73, 1.12)	0.362	229	1.55 (0.95, 2.53)	0.079^a^

SY = School year.

Odds ratios for each test represent odds of being in the lowest 15% of the test score.

a Significant difference between children of mothers who ate fish and those whose mothers did not.

b Adjusted for month of birth relative to school year, sex of child, maternal age, parity, maternal education, family adversity index, housing tenure, household crowding, prenatal life events, prenatal smoking, prenatal alcohol.

To determine whether the linear relationships analysed were masking threshold effects, the data were broken down into quintiles for the two maths and the science tests where interactions with fish eating were demonstrated (Appendix Table 2). These show no indication of a fall in test results at the highest levels of prenatal total blood mercury; indeed the reverse was true.

### 3.3 Sex differences

All analyses were repeated for boys and girls separately, but no differences were statistically significant (data not shown).

## 4 Discussion

This study, which is the largest single population study to date, has emulated the results from the Seychelles (Davidson et al., 2010, Davidson et al., 2011) by not being able to demonstrate any adverse scholastic test results with increasing prenatal total mercury levels. However, we have shown different associations between specific tests of mathematical and scientific understanding when the mother eats fish compared with those who do not.

To our knowledge no studies have looked at interactions of prenatal total mercury levels with maternal fish consumption apart from our own study of the child's IQ (Golding et al., 2017), and a similar study of cognitive function in the INMA study in Spain (Llop et al., 2016). Both showed a similar interaction, with significant differences between the positive association when the mother was a fish eater and a more negative association when she did not consume fish. No other studies have assessed possible interactions with scholastic ability. It is important that our finding of interactions specific to mathematics and scientific understanding be assessed in other studies.

We have concentrated on tests of mathematical and science reasoning designed particularly for the ALSPAC study and administered in schools. The results of these tests were used rather than the national tests as they were specifically designed to test mathematical reasoning. It has been shown that the maths tests, together with knowledge of arithmetic (as assessed in year 4), make independent contributions to children's achievement in mathematics in the national tests. Consequently, we chose to use both mental arithmetic and the mathematical reasoning tests (Nunes et al., 2009). Similarly, the science reasoning test was shown to be strongly predictive of the national science tests later in the child's school life (Bryant et al., 2015).

If there are differences between the children of fish and non-fish eaters, the key question concerns the possible causes of such an interaction. There are several possibilities. One concerns interactions with other features of the diet. We have shown elsewhere (Golding et al., 2013) that some dietary factors appear to be negative predictors of total blood mercury in our study population, including white bread, whole milk, sugar, French fries, baked beans, and meat pies/pasties. Consistent with these findings, Bates et al. (2007) reported negative associations between total blood mercury and white bread, whole milk, sugar, and French fries in a study of 1216 British adults 19–64 years of age. Other possible explanations relate to the various beneficial components of fish including iodine, omega-3 fatty acids, choline and vitamin D, all of which have been shown to be associated with improved cognitive abilities (Darling et al., 2017).

### 4.1 Strengths and weaknesses

Mercury levels were obtained from blood collected from the mother in the first trimester of pregnancy. This is more appropriate than mercury exposure based on the assumptions from intake of diet alone (e.g. the Norwegian Mother and Child Cohort Study: Vejrup et al., 2014). Nevertheless, it should be noted that the measures may not be representative of those found later in pregnancy, e.g. those obtained from maternal hair, cord blood or placenta.

All scholastic test results were obtained without knowledge of the level of exposure of the fetus to mercury or of whether the mother ate fish or not. Many of the tests were designed specifically to test different aspects of the child's ability.

The seafood eaten in the study area does not include sea mammals. This is likely to be important as sea mammals, such as whale, have far fewer beneficial nutrients than found in fish, and more pollutants such as PCBs; consequently studies including sea mammals such as that from the Faroes (Weihe et al., 1996) may be less likely to show any beneficial associations of mercury from seafood consumption.

In comparison with other studies, the sample we utilised was large. Nevertheless, as with all longitudinal studies, there was notable attrition among those where attendance at a clinic was necessary to obtain the results. We have shown elsewhere that this causes bias, with excess drop-out from young parents, smokers, those of lower educational achievement, and those living in rented accommodation (Gregg et al., 2005). However, for the tests undertaken in schools (maths, spelling and science), all children in a class were included, and the biases were more concerned with whether the child's family had left the study area and/or the attitude of the teacher. The fact that the results were broadly similar regardless of type of test adds validity to the findings of no adverse effects of maternal total mercury levels. The major disadvantage of the study design lies in the relatively small numbers of children in the study whose mothers did not eat fish during pregnancy.

## 5 Conclusion

The data analysed in this study have given no indication that there were any adverse effects of prenatal maternal total mercury levels on the scholastic abilities of the offspring. However, there was a suggestion that children whose mothers denied eating fish were less likely to do well in mathematics and scientific reasoning with increasing exposure to mercury than the child whose mother had eaten fish in pregnancy. Replication in other cohorts is needed, but meanwhile the recommendation to eat at least two portions of fish per week should be supported.

## Figures and Tables

**Appendix Table 1:** Mean and median total blood mercury (pg/l) in pregnancy for demographic variables

	N	Median	Mean (SD))	R^2^ (%)
**Maternal age (years)**				
<20	239	1.34	1.50 (0.74)	
20-24	813	1.57	1.79 (1.00)	
25-29	1531	1.90	2.11 (1.11)	
30-34	1019	2.12	2.29 (1.07)	
35+	311	2.18	2.44 (1.21)	4.87

**Parity (n)**				
0	1622	1.97	2.22 (1.24)	
1	1249	1.86	2.02 (0.95)	
2	540	1.86	2.02 (0.90)	
3+	239	1.68	1.86 (0.95)	0.96

**Maternal education**				
A (lowest)	673	1.54	1.75 (0.95)	
B	335	1.73	1.89 (1.01)	
C	1155	1.88	2.03 (1.03)	
D	802	2.05	2.29 (1.16)	
E (highest)	547	2.40	2.60 (1.18)	5.98

**Smoked mid-pregnancy**				
Yes	752	1.61	1.83 (0.97)	
No	2968	1.96	2.16 (1.11)	1.50
**Alcohol consumption (units) mid pregnancy** Not at all	1789	1.76	1.96 (1.05)	
<1/week	1228	1.95	2.17 (1.07)	
1-6 / week	550	2.09	2.28 (1.06)	
1+ / day	65	1.94	2.39 (1.56)	1.44

**Housing tenure**				
Owned/mortgaged	2695	2.00	2.20 (1.10)	
Council rented (public	570	1.52	1.71 (0.96)	
housing) Other	444	1.79	2.02 (1.18)	2.61

**Appendix Table 2:** Mean (SE) maths and science test scores according to prenatal total mercury levels divided at the 20^th^, 40^th^, 60^th^, 80^th^, and 90^th^ centiles

Prenatal total blood mercury (^g/l)	N		Test score (mean (SE))	
		Maths SY6	Maths SY8	Science SY6
*All children*				
<1.28	388	18.04 (0.35)	21.64 (0.59)	5.43 (0.12)
1.28-1.68	432	18.82 (0.35)	22.19 (0.55)	5.80 (0.13)
1.69-2.10	393	19.52 (0.36)	22.68 (0.52)	5.99 (0.14)
2.11-2.74	409	19.53 (0.36)	24.50 (0.47)	6.28 (0.13)
2.75-3.39	217	20.16 (0.47)	25.25 (0.68)	6.21 (0.17)
>3.39	202	21.49 (0.49)	25.80 (0.62)	6.91 (0.18)
*P for trend*		*<0.0001*	*<0.0001*	*<0.0001*

*Children whose mothers ate fish*				
<1.28	206	18.17 (0.49)	22.57 (0.75)	5.71 (0.17)
1.28-1.68	309	19.45 (0.40)	23.08 (0.60)	6.07 (0.14)
1.69-2.10	329	20.04 (0.38)	22.88 (0.58)	6.13 (0.15)
2.11-2.74	358	19.94 (0.38)	24.41 (0.51)	6.42 (0.14)
2.75-3.39	188	20.51 (0.48)	25.59 (0.68)	6.29 (0.18)
>3.39	177	21.86 (0.52)	25.89 (0.64)	7.11 (0.19)
*P for trend*		*<0.0001*	*0.0005*	*<0.0001*

*Children whose mothers ate no fish*				
<1.28	128	18.42 (0.61)	21.19 (1.18)	5.30 (0.20)
1.28-1.68	69	18.57 (0.87)	19.52 (1.65)	5.79 (0.34)
1.69-2.10	34	16.47 (1.26)	22.71 (1.53)	5.06 (0.52)
2.11-2.74	24	17.25 (1.13)	23.42 (1.84)	5.45 (0.50)
2.75-3.39	10	16.80 (2.73)	26.25 (1.03)	4.73 (0.92)
>3.39	10	16.90 (2.42)	29.00 (3.00)	5.30 (0.87)
*P for trend*		*0.646*	*0.308*	*0.677*

SY, school year.

## Appendix B Supplementary data

Details of the scholastic tests used. Image 1
