# Supplementary material for: Total mercury exposure in early pregnancy has no adverse association with scholastic ability of the offspring particularly if the mother eats fish
Source: Environ Int. 2018 Jul;116:108–15. doi: 10.1016/j.envint.2018.03.024 (PMC5993870; doi:10.1016/j.envint.2018.03.024)
Supplement: Supplementary file 1 — Details of the scholastic tests used. [file mmc1.pdf]

Appendix B Supplementary Material

Details of the scholastic tests used

Tests of spelling ability

At both the 7-year and the 9-year clinics, a spelling test was administered immediately after the reading session. A total of 15 words were chosen specifically for each age group after piloting on several hundred children by Peter Bryant and Terezinha Nunes of the Department of Education at the University of Oxford. The spellings involved regular and irregular words of different frequencies. They were given in order of increasing difficulty as identified from the pilot studies. For each, the word was read out aloud to the child, and then within a specific sentence incorporating the word, and then alone again. The child was asked to write down the spelling even if he/she thought they were just guessing at the spelling. The spelling score was the number of words spelt correctly (range 0–15).

The spelling scores for each age included those who stopped early (usually because they had reached the limit of their ability). The scores were not normally distributed but did not have ceiling effects. Scores were available for 8031 and 7640 of the 7- and 9-year-old children, respectively.

Tests of reading ability

(a) Word reading

For the reading assessment at age 7 years, the basic reading subtest of the WORD (Wechsler Objective Reading Dimension) was used for all children attending the ALSPAC clinic. Pictures and words were used to assess decoding and word reading (Rust et al. 1993).

The child was shown a series of four pictures by trained psychologists. Each picture had four short simple words underneath it. The child was asked to point to the word which had the same beginning or ending sound as the picture. This was then followed by a series of three pictures, each with four words beneath, each starting with the same letter. The child was asked to point to the word that correctly named the picture. Finally the child was asked to read aloud a series of 48 unconnected words which increased in difficulty. If the child read the word incorrectly but pronounced it in a way that was phonetically plausible, this was also noted for each word. The reading task was stopped after the child had made six consecutive errors. 8070 children completed the task; the score was the number of items the child responded to correctly. It ranged from 0 to 54, with mean 28.1 (SD 9.44).

At age 9 years, the child was asked to read aloud ten real words, followed by ten non-words. Both the words and non-words were selected from a larger selection of words taken from previous research (Nunes, et al. 2003). The two sets of words were specifically chosen for this study by Nunes and Bryant (more details of the non-word test is given in the description of phonological awareness below).

The test–retest reliability of the word reading is 0.8, and the scale has a correlation of 0.85 with the Schonell Word Reading Task **(**Schonell and Goodacre, 1971**)**. Under test conditions, the child was shown each word in turn and asked to read the words out loud. The word reading score was calculated as the number of words read correctly (range 0–10). A total of 7657 children had a score which ranged from 0 to 10, but the distribution demonstrated a ceiling effect.

(b) Reading comprehension

The revised Neale Analysis of Reading Ability (NARA II) (Neale, 1997) was used to assess the child's reading skills and comprehension at age 9 years. This test is suitable for children between the ages of 6 and 12 years with a standard assessment time of 20 minutes. It was administered by trained psychologists using Form II. The testing took place in a quiet room. Wherever possible, parents were asked not to accompany their child into the testing room to minimize distractions and interruptions.

The child was first given a practice story; the same structure for testing was used for this and all subsequent test passages. A booklet was used from which each child read a passage, they were then asked a series of questions about the content of the story they had just read. The tester recorded the time (in seconds) it took the child to read the passage. Any errors made by the child during reading were noted on the data sheet. The child was prompted by the tester if they: (a) mispronounced a word; (b) substituted a word; (c) refused to say a word; (d) made an addition (only if it altered the meaning of the story); (e) made an omission; or (f) reversed a word.

Administration of the test was undertaken following the instruction manual. If the child made more than 17 errors on the practice passage, the tester did not ask the child the comprehension questions but moved straight on to the level one story. All other children moved on to the level two story unless the tester felt that they had difficulty with reading the practice passage. If the child made less than three errors on the level two story the tester moved on to level three. If, however, the child made three or more errors on level two, the comprehension questions were administered but the tester moved down to the level one story (only moving on to level three if the child completed level one within the permissible number of errors). For the remaining test passages the child was not asked the comprehension questions if they made more than 16 errors (20 on level six) and the session was ended.

The comprehension questions were asked as soon as the child had finished reading. For each question the child was given 10–12 seconds to respond; they could refer to the text to assist them.

(c) The comprehension score

The raw comprehension score was obtained by summing the number of correct answers the child gave for each passage. If the permissible number of errors was exceeded for the final passage, the comprehension questions were not asked so no score was given for that passage. The conversion of the raw score to a score standardised for age used the test authors' criteria**.** It was approximately normally distributed and available for 6943 children. It should

be noted that 48 children were unable to attempt the test and have been excluded. However when examining the outcome of reading impairment, we have included these 48 children in the impaired group.

(d) Reading speed

Using the times taken for the child to read each passage, a speed rate of words per minute was computed for each child. This was based on only those passages read where no more than 16 errors were made (20 for passage 6) and was created as follows:

Rate per minute = Total no. words read × 60 / Total time taken (sec)

The reading speed standardised for age was approximately normally distributed with mean 105.1 (SD 12.6).

(e) Reading accuracy

The accuracy score was computed as the total number of errors made by the child in all the passages that they read, such that the higher the score the worse the accuracy. This was standardised for age at testing using the author's criteria and is reasonably normally distributed (n = 6943). It had a mean of 103.6 (SD 13.7).

(f) Reading fluency or sight-word efficiency

At the 13 year assessments, a word reading task (the TOWRE task (Torgesen et al. 1999)) was given to the study children. It provides a test of sight-word efficiency. A list of 104 words was given to the child to read, and the number read accurately within 45 seconds was recorded. The score identifying the number of words read in the time (but not the accuracy) was approximately normally distributed.

Tests of understanding of phonemes

(a) Phoneme deletion

The Phoneme Deletion task used at age 7 years after the reading and spelling assessments was the Auditory Analysis Test (Rosner and Simon, 1971). The task involved asking the child to repeat a word and then to say it again but with part of the word (a phoneme or number of phonemes) removed. For example, the child was asked to say 'sour' and then say it again without the 's' to which the child should respond 'our'. There were seven categories of omission: (a) omission of a first, (b) a medial or (c) a final syllable; (d) omission of the initial; (e) omission of the final consonant of a one syllable word; (f) omission of the first consonant or (g) consonant blend of a medial consonant.

Words from the different categories were mixed together but were placed in order of increasing difficulty. 8132 children were tested with the 40 words: the Phoneme Deletion

score ranges from 0 to 40, mean 20.0 (SD 9.6). The higher the score the more proficient the child.

(b) Phonemic decoding efficiency (non-word reading):

As noted earlier the 9-year-old child was asked to read aloud ten real words and 10 non-words. The non-word reading task has a test-retest reliability of 0.73 and correlations of 0.73 and 0.77 with reading and spelling tasks given four months later (Nunes et al. 2003). It was emphasised to the child that because they were invented the child would not recognise them as real words. The child was asked to read all the words in the way that they thought they should be read, even if they were guessing. The tester recorded whether the child pronounced the word correctly or incorrectly. The number of non-words read correctly was completed by 7643 children. The distribution ranged from 0 to 10 and was approximately normal with mean of 5.2 (SD 2.4).

Tests of mathematical ability

The development of numeracy and mathematical skills is made up of several components which are built on in hierarchical ways over time (Duckworth, 2008). Even before children enter formal schooling they intuitively start to piece together basic mathematical concepts such as relative size and counting. Much of the research on the development of mathematical skills has focussed on arithmetic or word problem solving but little is known about influences on the general course of mathematics performance in non-selected populations.

Mathematics tested by ALSPAC in schools

(a) Mathematical reasoning tests

To obtain data appropriate to the aims of ALSPAC, various advisors were asked for their opinions over time. These included expert researchers including Terezhina Nunes and Peter Bryant, as well as representatives of the local Avon education authorities and other expert contributors. The result was the recommendation to use tests devised for the study by Nunes and Bryant which would ensure the measurement of mathematical reasoning. The aim of these Mathematical Reasoning tasks was to assess children's understanding and use of quantitative relations, to solve mathematical problems. They designed two different Mathematical Reasoning tasks. One, containing 17 items, was given to school-children in Year 4 (N= 5275, mean age 8 years 9m). The other, containing 35 items, was given to children in Year 6 (N= 7981, mean age 11 years 2m) and again in Year 8 (N= 2755, mean age 12 years 8m).

The aim of these tasks was to assess children's reasoning about quantities and the relations between quantities in mathematical problems independently of their computation skills. None of the items in these tests contained difficult calculations; the children had to reflect on the relations between quantities in each item to decide how to solve the problem. All the items were presented with the support of drawings; the children could use counting to

solve many of the problems if they did not know the number facts that might be used in the solution. All the problems were presented orally by the teachers to avoid an undue influence of reading difficulties on the children's performance (Nunes et al. 2009).

Three types of item were included in the Year 4 Mathematics Reasoning Task: additive reasoning items about quantities, additive reasoning items about relations, and multiplicative reasoning items about quantities. The assessments used in Years 6 and 8 included six types of item: additive reasoning items about quantities; additive reasoning items about relations; multiplicative reasoning items about quantities; multiplicative reasoning items involving relations (i.e. proportions); items about spatial reasoning and items about fractional quantities.

Analyses of their internal consistency using Cronbach's α showed that on all three occasions the mathematics reasoning tasks had good levels of inter-item reliability: 0.74 at Year 4 (N=5275), 0.89 at Year 6 (N=7881) and 0.91 at Year 8 (N=2755). This high internal consistency justifies the addition of all the items in each school year into single scores.

(b) Mental arithmetic

Mental Arithmetic was measured as part of the WISC verbal intelligence tests at age 8 years. The raw scores at age 8 years were measured using alternate questions as for the WISC test in general (Golding et al. 2017). The data were approximately normally distributed.

Scientific reasoning

A test of scientific reasoning was developed specifically for the study by Nunes and Bryant. The aim was to measure children's understanding that in a properly controlled scientific comparison, one variable is tested at a time while other variables are held constant. The test was administered in school year 6 (age 11–12 years) by the class teacher. The pupils' scores successfully predicted their later progress in science even after allowance was made for age and IQ (Bryant et al. 2015).

References

Bryant P, Nunes T, Hillier J, Gilroy C, Barros R. The importance of being able to deal with variables in learning science. *International Journal of Science and Mathematics Education*. 2015;13(1):145-63. Doi. 10.1007/s10763-013-9469-x

Duckworth, K. (2008). *The influence of context on attainment in primary school: Interactions between children, family and school contexts (Wider Benefits of Learning Research Report No. 28)*. Centre for Research on the Wider Benefits of Learning, Institute of Education, University of London. Doi. None

Neale MD (1997). Neale Analysis of Reading Ability – Revised. Windsor: NFER-Nelson.

Nunes T, Bryant P, Olsson J. Learning morphological and phonological spelling rules: An intervention study. *Scientific Studies of Readi*ng 2003; 7: 289-307. Doi. 10.1207/S1532799XSSR0703_6

Nunes T, Bryant P, Sylva K, Barros R. (2009) Development of maths capabilities and confidence in primary school. Research Report no. DCSF-RR118; University of Oxford. Pp1-96. ISBN 978 1 84775 457 8

Rosner J & Simon DP. (1971) The Auditory Analysis Test An Initial Report. *Journal of Learning Disabilities*. 4(7):384-392. Doi. 10.1177/002221947100400706

Rust J, Golombok S, Trickey G. (1993). WORD: Wechsler objective reading dimensions manual. Psychological Corporation, London.

Schonell F, Goodacre E (1971). The psychology and teaching of reading. London: Oliver and Boyd.

Torgesen JK, Wagner R & Rashotte C. (1999) TOWRE–2 Test of Word Reading Efficiency. Mind Resources Inc. Austin, TX: Pro-Ed.
